# Integrating artificial intelligence and in-silico modeling to redefine therapeutic strategies for peritoneal seeding: A disulfiram repurposing concept

**DOI:** 10.12669/pjms.42.6.16393

**Published:** 2026-06

**Authors:** Jieun Yang, Ji Eun Park, Sanghoon Han

**Affiliations:** 1 Jieun Yang, Department of Internal Medicine, Jeju National University Hospital, Jeju National University College of Medicine, Republic of Korea; 2 Ji Eun Park, Department of Internal Medicine, Jeju National University Hospital, Jeju National University College of Medicine, Republic of Korea; 3 Sanghoon Han, Department of Internal Medicine, Jeju National University Hospital, Jeju National University College of Medicine, Republic of Korea

Despite advances in systemic chemotherapy, peritoneal metastasis remains clinically challenging due to limited drug delivery across the blood-peritoneal barrier—a biological “deadlock” that prevents systemic agents from reaching therapeutic concentrations within the abdominal cavity. We propose a paradigm-shifting concept by integrating AI-assisted in-silico predictive analysis to redefine the therapeutic potential of Disulfiram (DSF).

Our computational model utilized high-resolution molecular docking and dynamic simulations to analyze the delivery-target interface. Specifically, this in-silico methodology confirmed a superior binding affinity of the DSF-copper complex to the NPL4 zinc-finger domain (a key protein in the p97 segregase pathway), which induces cancer cell apoptosis via proteotoxic stress.^1^ As highlighted in recent AI-driven drug discovery frameworks^2^, addressing the delivery-target interface through structural bioinformatics is crucial to overcome previous systemic failures. Contrary to being a mere carrier, DSF acts as a potent active chemotherapeutic agent when its lipophilic properties are leveraged for sequestration into lipid-based nano-carriers (e.g., Lipiodol droplets).

**Fig.1:**
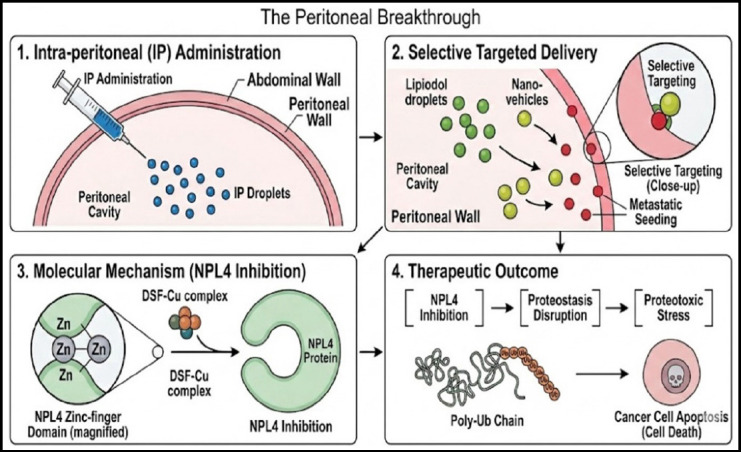
AI-driven Disulfiram (DSF) repurposing strategy for peritoneal metastasis. (A) In-silico methodology: High-resolution molecular docking and dynamic simulations confirm the binding of the DSF-copper complex to the NPL4 zinc-finger domain. (B) Delivery mechanism: Lipophilic DSF is encapsulated into nano-vehicles and lipid droplets (e.g., Lipiodol) to ensure sustained drug release and sequestration within the abdominal cavity. (C) Clinical platform: The formulation is delivered via Pressurized Intra-Peritoneal Aerosol Chemotherapy (PIPAC), utilizing physical pressure to overcome the blood-peritoneal barrier and maximize drug penetration into peritoneal nodules.

This strategy is optimized for localized delivery via established platforms such as Intra-Peritoneal (IP) administration and Pressurized Intra-Peritoneal Aerosol Chemotherapy (PIPAC)—a technique that uses physical pressure to enhance drug penetration into peritoneal nodules.^3^ By directly coupling AI-derived target engagement with IP/PIPAC platforms^4^,^5^, this strategy has the potential to deliver higher intraperitoneal drug intensity with lower systemic toxicity in real-world clinical practice. Shifting toward this AI-integrated framework provides a rational basis for future clinical trials aiming to overcome refractory peritoneal carcinomatosis.
